# Adverse Respiratory Events After Removal of Laryngeal Mask Airway in Deep Anesthesia Versus Awake State in Children: A Randomized Trial

**DOI:** 10.7759/cureus.24296

**Published:** 2022-04-19

**Authors:** Shemila Abbasi, Khalid M Siddiqui, Muhammad Qamar-ul-Hoda

**Affiliations:** 1 Anaesthesiology, The Aga Khan University Hospital, Karachi, PAK

**Keywords:** respiratory complications, awake technique, planes of anesthesia, laryngeal mask airway (lma), adverse respiratory events

## Abstract

Background

The advent of the laryngeal mask airway (LMA) has reduced respiratory events in comparison to the conventional endotracheal tubes. Any manipulation under a light plane of anesthesia predisposes to increased airway sensitivity followed by adverse events. The reduced airway sensitivity in the deeply anesthetized state makes LMA removal feasible. In the past, the respective advantages and disadvantages of extubation in two planes of anesthesia have led to conflicting results. The primary objective of this study is to compare the incidence of adverse respiratory events at the time of LMA removal, in deeply anesthetized and awake groups. Our secondary objective was to record the management of complications.

Materials and methods

We conducted a prospective randomized control trial in 106 American Society of Anesthesiologists (ASA) I and II patients undergoing lower umbilical surgeries over a period of one year. The demographic details and intraoperative and postoperative variables, i.e., airway obstruction, laryngospasm, peripheral oxygen desaturations, cough, straining and vomiting, along with corrective measures were recorded by the primary research assistant in both groups. Regarding the management of peripheral oxygen desaturation (less than 90%), airway obstruction, and laryngospasm, 100% fractional inspired oxygen support and chin lift/jaw thrust were used.

Results

The average age was 32.58±15.81 months. The demographic characteristics of the patients were not significant between the two groups. The rate of adverse respiratory events like laryngospasm and airway obstruction was relatively high in the deep group but not statistically significant between the groups. A total of 7 (6.6%) patients had laryngospasm, 21 (20%) had airway obstruction, 16 (15%) had a cough and 11 (10%) patients had observed peripheral oxygen desaturation (less than 90%) between both groups.

Conclusion

We concluded that adverse respiratory events could happen in both awake and deep planes of anesthesia after the removal of LMA in children. Furthermore, both techniques have an acceptably low frequency of complications, and it does not affect the current clinical practice.

## Introduction

The introduction of the laryngeal mask airway (LMA) has changed the face of airway management in modern-day anesthesia [1]. Its advent has reduced adverse respiratory events in comparison to the conventional endotracheal tubes [2], and it is now a part of the management of a difficult airway algorithm [3]. Despite the expanded role and innovations in its design, there remain some unanswered questions about the best timing of LMA removal to reduce adverse respiratory events [4]. During the use of LMA, any manipulation under a light plane of anesthesia predisposes to increased airway sensitivity and possible incidence of retching, straining or even laryngospasm [5]. It is still debatable which plane is better to avoid these untoward effects [6].

The reduced airway sensitivity in the deeply anesthetized state makes removal of LMA more feasible, but the decreased muscle tone can lead to obstruction of the airway [7]. Similarly, an awake patient who is fully conscious, alert, aware and regained his/her protective airway reflexes can maintain his/her airway, but this awareness renders the airway more reactive to a stimulus and hence increased the chances of coughing [8]. The primary goal of this study is to evaluate the occurrence of adverse respiratory events, after LMA removal at the end of the surgery, in the awake and deep plane of anesthesia in children. Our secondary objective is to record the management strategies of respiratory complications in both groups.

## Materials and methods

**Figure 1:**
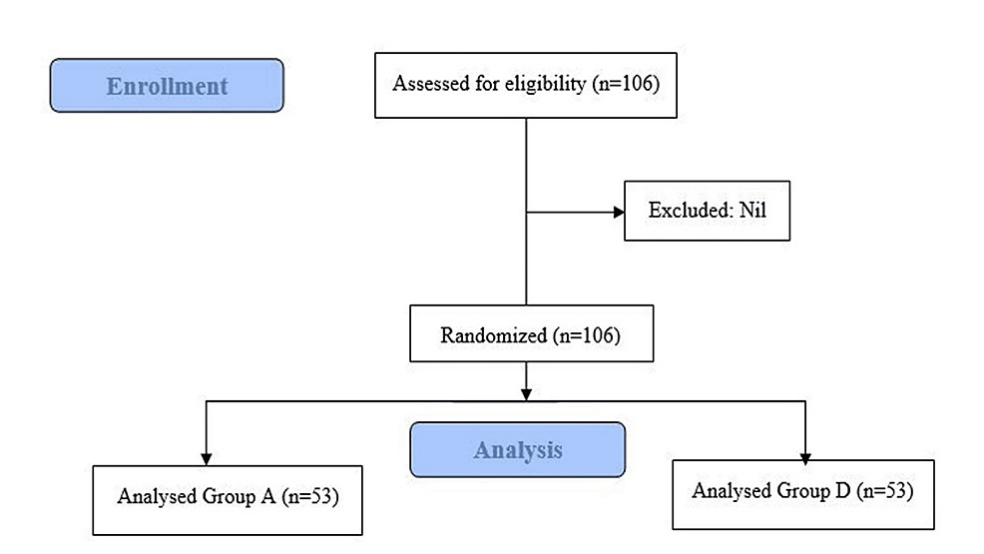
CONSORT (Consolidated Standards of Reporting Trials) Flow diagram of the study

We performed a single-center, prospective, randomized trial in a university hospital. This study was approved by the Ethical Review Committee (Reference: ERC no: 4767-Ane-ERC-17) of the Aga Khan University Hospital, and written informed consent was taken from all parents/guardians of the participating patients. The study was conducted in the main operating suite from August 2017 to July 2018. Subjects were randomly divided into two groups (group A=awake, group D=deep) by a sealed envelope technique. The sample size calculation was based on previous data [8]. Fifty-three subjects per arm were recruited to detect a 50% difference in the incidence of adverse respiratory events at an alpha of 0.05 and beta of 0.80. A reduction in the incidence of adverse respiratory events by 50% was considered clinically significant. A total of 106 patients were enrolled, with a nonprobability consecutive sampling technique. A total of 53 patients in each group with American Society of Anesthesiologists (ASA) I and II, aged more than two and less than 16 years, surgery duration up to 90 minutes and all elective infraumbilical surgeries were included. Patients with a full stomach or emergency surgeries, history of upper or lower respiratory tract infection within the last four weeks, gastroesophageal reflux disease, reactive airway disease, trauma that happened during LMA insertion or more than two attempts of LMA insertion were excluded from the study (Figure 1).

In group A, LMA was removed when patients were awake that means fully conscious, alert, aware and have regained their protective airway reflexes with spontaneous ventilation, while in group D, LMA was removed when patients were in a deep plane of anesthesia. Anesthesia protocols for all the subjects were standardized. All the patients included in the study were premedicated with oral midazolam (0.5 mg/kg), an hour before the procedure. Standard monitoring was applied including ECG, noninvasive blood pressure (NIBP), end-tidal CO_2_ and pulse oximeter to monitor oxygen saturation. Induction was done with propofol 1.5 to 2.5 mg/kg if the intravenous cannula is already placed, or inhalational induction with sevoflurane with oxygen was done if the intravenous cannula is not placed. Single-use LMA (manufactured by Ambu® AuraOnce™, Xiamen, Fujian, China) size was calculated by the patient’s weight before insertion. Insertion and removal of LMA were only done by a primary anesthesiologist who has at least two years of anesthesia experience. Once LMA has been placed and fully inflated, correct positioning was ensured based on chest expansion, end-tidal carbon dioxide concentration and peripheral oxygen saturation. After induction, the depth of anesthesia was maintained by isoflurane and nitrous oxide with fractional inspired oxygen (FiO_2_) of 40%, maintaining a minimum alveolar concentration (MAC) of 1.2. Analgesia was provided with the caudal block (ropivacaine 1 ml/kg, 0.25%), supplemented with intravenous paracetamol 10 mg/kg. Intravenous morphine 0.1 mg/kg was used as rescue analgesia. Peripheral oxygen saturation, end-tidal CO_2_, anesthetic concentration and minimum alveolar concentration (MAC) were measured on an S/3 Multipurpose monitor (Datex-Ohmeda Division, Helsinki, Finland).

After the surgery in group A, LMA was left in situ while isoflurane was turned off, and it was ensured that the patients remained unstimulated till they regained consciousness. In group D, at the time of emergence, nitrous oxide was turned off and isoflurane MAC with 100% oxygen was maintained at 1.3 for five minutes. LMA was removed in an undeflated state in both the groups along with suctioning if required. Patients were followed for any adverse respiratory events. In case of airway obstruction, chin-lift/jaw-thrust maneuvers were applied followed by insertion of the correct size of the oropharyngeal (Guedel) airway (manufactured by Well Lead Medical Co. Ltd. Panyu, Guangzhou, China) and placement of the patient in the left decubitus position. Any untoward event such as laryngospasm or peripheral oxygen desaturation below 95% was intended to manage with positive pressure ventilation followed by appropriate pharmacological interventions.

Propofol 0.5 mg/kg and succinylcholine 0.5 mg/kg were used to reinsert LMA or endotracheal intubation to protect the airway. Adverse respiratory events were managed by a primary anesthesiologist/consultant. Emergency intubation equipment was also made available during the study. Regarding the management of peripheral oxygen desaturation, we intended to deliver 100% fractional inspired oxygen support, chin lift/jaw thrust, use of Guedel airway, positive pressure ventilation, laryngeal mask reinsertion and finally endotracheal intubation if needed.

Intraoperative data collection was done at the time of induction, at the time of removal of LMA and before shifting the patient to the postanesthesia care unit (PACU). Data collection was continued in PACU to observe any sequel of an adverse respiratory event that occurred in the operating room. Variables were measured including peripheral oxygen saturations (SpO_2_) and minimum alveolar concentration (MAC) of inhaled anesthetic. All variables were observed at the time of insertion and removal of LMA including any upper airway trauma, the number of attempts at insertion, airway obstruction (including laryngospasm), peripheral oxygen desaturations, cough, straining and vomiting, along with the measures taken to correct them. Before moving the patients to PACU, final documentation was also done.

Statistical analysis

The statistical analysis was performed using statistical packages for social science version 21 (SPSS Inc., Chicago, IL). Frequency and percentage were computed for gender, ASA status and adverse respiratory event (complication). The median interquartile range (IQR) was reported for non-normal distribution and analyzed by the Shapiro-Wilk test. Chi-square test was applied to compare adverse respiratory events between groups A and D. p<0.05 was considered significant. Stratification was done to control effect modifiers like age, gender and ASA status to observe outcomes.

## Results

Total patients included in the study were 106, out of which 99 were males and 7 were females, and there were no dropouts. The average age of the patients was 32.58±15.81 months. Other demographic and clinical characteristics of the patients were comparable in both groups as shown in Table 1.

**Table 1:** Demographic and Clinical Characteristics of Patients ASA: American Society of Anesthesiologists, BP: blood pressure.

Variables	Awake n=53	Deep n=53	p-value
Age (months)	25.00 (21-45)	30.00 (30-48)	0.94
Gender			
Male	48 (90.6%)	51 (96.2%)	0.241
Female	5 (9.4%)	2 (3.8%)	
ASA status			
I	44 (83%)	47 (88.7%)	47 (88.7%)
II	9 (17%)	6 (11.3%)	6 (11.3%)
Duration of surgery (per min)	51.62±20.95	46.30±16.71	0.151
Baseline heart rate (per min)	119.21±17.76	118.11±20.55	0.768
Peripheral oxygen saturation	98.92±0.70	98.75±0.97	0.307
Systolic BP (mmHg)	79.88±13.44	83.62±12.97	0.148
Diastolic BP (mmHg)	43.2±9.09	45.09±10.10	0.532

The incidence of adverse respiratory events including laryngospasm, airway obstruction followed by cough and peripheral oxygen desaturation was nonsignificant between the two groups (Table 2). A total of 11 patients had an episode of desaturation less than 90%, managed by the provision of inspired oxygen concentration of 100% and jaw-thrust/chin-lift maneuver. Peripheral oxygen desaturation and cough were two adverse respiratory events observed in PACU as well which were also nonsignificant.

**Table 2:** Comparison of Adverse Respiratory Events After LMA Removal Between Awake and Anesthetize Patients Within Operating Room and Recovery Room LMA: laryngeal mask airway.

Operating room					Recovery room			
	Awake n=53	Deep n=53	Total n=106	p-value	Awake n=53	Deep n=53	Total n= 106	p-value
Laryngospasm	2 (3.7%)	5 (9.4%)	7 (6.6%)	0.241	0	0	0	-
Airway obstruction	3 (5.6%)	18 (33.9%)	21 (20%)	0.367	0	0	0	-
Cough	8 (15.1%)	8 (15.1%)	16 (15%)	0.999	1 (1.9%)	5 (9.4%)	6	0.205
Peripheral oxygen desaturation (<95%)	5 (9.4%)	6 (11.3%)	11 (10%)	0.587	3 (5.7%)	4 (7.5%)	7	0.999
Straining/biting	1 (1.9%)	2 (3.8%)	3 (2.8%)	0.558	0	0	0	-
Vomiting	1 (1.9%)	0 (0%)	1 (0.9%)	0.999	0	0	0	-

Management of airway obstruction in both groups is shown in Table 3. All patients were managed successfully, and no endotracheal intubation was required in any of the groups.

**Table 3:** Management of Peripheral Oxygen Desaturation or Airway Obstruction

Management	Awake n=53	Deep n=53	p-value
100% fractional inspired oxygen support	14 (26.4%)	13 (24.5%)	0.824
Chin lift/jaw thrust	15 (28.3%)	12 (22.6%)	0.504
Use of Guedel airway	4 (7.5%)	2 (3.8%)	0.678
Left decubitus position	1 (1.9%)	0 (0%)	0.999
Positive pressure ventilation	2 (3.8%)	1 (1.9%)	0.558
Laryngeal mask reinsertion	5 (9.4%)	2 (3.8%)	0.241
Endotracheal intubation	0	0	-

## Discussion

A majority of patients in our study are males. Other demographic and clinical characteristics of the patients were comparable in both groups. The overall incidence of laryngospasm and airway obstruction was more in group D but clinically insignificant. The timing of laryngospasm was noted just after the removal of LMA.

Respiratory adverse events are always a major concern for anesthesiologists in every age group, but their importance has increased when it comes to the pediatric airway. The time of induction and intubation and the emergence and removal of airway devices are two major phases of anesthesia that required special attention. The emergence in children is more challenging because of the transition of caregivers as well as a new area. There are pros and cons of awake versus deep sleep in a child. In the current study, we tried to observe both planes to assess adverse respiratory events and found insignificance in both groups. One of the earlier works in this regard was by Laffon et al. [9] who tried to demonstrate the decreased incidence of adverse respiratory events in the deep state of anesthesia. However, their claims were soon rejected as it became apparent that the medical practitioners used in the study were inexperienced in LMA handling [10].

Previously, Dolling and colleagues [11] have recommended that LMA should be removed while the patient is awake to reduce the adverse respiratory events. The argument behind this conclusion is related to the sharing of airways between surgeons and anesthesiologists. However, a recent meta-analysis comprised 17 randomized trials (1,881 patients) that compared airway problems in pediatric patients after general anesthesia between awake and deep plane extubation. Meta-analysis shows that in pediatric patients, deep extubation decreased the risk of overall complications such as cough and desaturation relative to awake extubation. However, it also emphasizes that deep extubation increases the risk of airway obstruction when compared to awake extubation, and there was no difference in the risk of laryngospasm between awake and deep extubation [12]. Our study also brings the same trends in terms of airway complications.

Oxygen desaturation was the most significant adverse respiratory event, and we also observed other complications such as airway obstruction, cough and laryngospasm specifically. A Cochrane Database of Systematic Review on early versus late removal of the laryngeal mask airway (LMA) had concluded that the risk of desaturation is very low in either part of the intervention [13]. This study also reflects the same trend in terms of oxygen desaturation in both groups.

Airway obstruction can happen due to malpositioning, biting or kinking on the tube; obstruction by the epiglottis; and laryngospasm. A light plane of anesthesia can also lead to laryngospasm and airway obstruction in children. Park and colleagues [14] did a randomized control trial on 92 ASA I and II children and found that the rate of airway-related complications was significantly less in the anesthesia versus awake group (4.8% versus 37.2%, p=0.001). In the current study, no difference was noted for upper airway obstruction between groups. On the other hand, Thomas-Kattappurathu and colleagues [15] conducted a randomized control trial on 216 ASA I and II patients aged one to 16 years and looked for the best position and depth of anesthesia for removal of LMA and found significant risk during LMA removal in deeply anesthetized patients in the supine position.

Besides airway obstruction, laryngospasm is also a crucial complication while removing LMA. The incidence of laryngospasm is reported to be 0.78%-5% after removal of LMA irrespective of the plane of anesthesia [16]. Children with an active upper respiratory tract infection (URTI), those receiving airway surgery or whose anesthesia was supervised by less experienced anesthesiologists were more likely to have laryngospasm. Michel et al. [17] studied the risk of perioperative respiratory adverse events (PRAE) in patients anesthetized in the presence of URTI and found it close to 30%. In view of their findings, they concluded that current rescheduling indications should be questioned, and further medical and organizational strategies should be investigated to reduce PRAE in children with URTI. Every day, many of us face the dilemma of a child with a URTI, and therefore, we must determine whether to proceed with surgery or postpone it for an extended period of time. Unfortunately, in a limited-resource setting, these things are overlooked on many occasions and the decision often rests on our individual comfort level in managing predictable complications.

On the contrary, Sinha and Sood [18] also looked at airway complications after removal of LMA in a deep anesthesia plane and awake group in a prospective single-blind study of 120 children of ASA I or II, one to eight years of age and undergoing lower abdominal surgeries. They found a complication rate of 35% in awake and 6.6% in deep groups. The difference in anthropometry and body composition of South Asian and European/North American children is an established fact [19], and other bases like hyper-reactive airway, respiratory tract infection and environmental pollution are more prevalent in this region [20]. Therefore, we expected to have a major difference in outcomes, but our study did not prove any difference.

The isoflurane 1.2% was used for the maintenance of anesthesia due to its cost-effectiveness. The isoflurane is comparatively more irritable than sevoflurane. On the other hand, sevoflurane can reduce airway irritability but may result in upper airway obstruction. Previous studies established the fact that both isoflurane and sevoflurane do not significantly affect adverse airway events after LMA removal and are suitable for day-care anesthesia [21,22]. The pivotal point of the study was to discover a safe technique for LMA removal. In this study, airway obstruction and laryngospasm were well managed by the slight chin or jaw lifting and did not lead to oxygen desaturation at a significant level. 

Removal of the LMA before or after the return of airway reflexes resulted in a similar incidence of postoperative airway problems. However, comparing the techniques between the two groups, we also noticed more time spent in the operation room in the awake group. The estimated time in the awake group was higher, whereas using the technique of removal of LMA in a deep plane can save more time during emergence. The observer’s bias is one of the limitations of our study as it was impossible to do blinding between deep and awake patients during LMA removal. However, by recording the respiratory problems by an observer different from the primary anesthesiologist, we tried to eliminate personal bias.

## Conclusions

Based on our study findings, we concluded that after removal of LMA, adverse respiratory events could happen in both awake and deeply anesthetized planes. No technique has its unique pros and cons, and based on the previous literature and the current study findings, we cannot recommend one method of LMA removal over the other. Both have an acceptably low frequency of complications, and it would not affect the current clinical practice.
